# Effects of common germline genetic variation in cell cycle control genes on breast cancer survival: results from a population-based cohort

**DOI:** 10.1186/bcr2100

**Published:** 2008-05-28

**Authors:** Elizabeth M Azzato, Kristy E Driver, Fabienne Lesueur, Mitul Shah, David Greenberg, Douglas F Easton, Andrew E Teschendorff, Carlos Caldas, Neil E Caporaso, Paul DP Pharoah

**Affiliations:** 1 Department of Oncology, University of Cambridge, Strangeways Research Laboratory, Worts Causeway, Cambridge, CB1 8RN, UK; 2 Division of Cancer Epidemiology and Genetics, National Cancer Institute, National Institutes of Health, Executive Plaza South, Rm 7002, 6120 Executive Blvd, Rockville, MD, 20852, USA; 3 Eastern Cancer Registration and Information Centre, Unit C-Magog Court, Shelford Bottom, Cambridge, CB22 3AD, UK; 4 Cancer Research UK Genetic Epidemiology Unit, Department of Public Health and Primary Care, Strangeways Research Laboratory, Worts Causeway, Cambridge, CB1 8RN, UK; 5 Cancer Research UK, Cambridge Research Institute, and Department of Oncology, University of Cambridge, Li Ka Shing Centre, Robinson Way, Cambridge, CB2 0RE, UK

## Abstract

**Introduction:**

Somatic alterations have been shown to correlate with breast cancer prognosis and survival, but less is known about the effects of common inherited genetic variation. Of particular interest are genes involved in cell cycle pathways, which regulate cell division.

**Methods:**

We examined associations between common germline genetic variation in 13 genes involved in cell cycle control (*CCND1*, *CCND2*, *CCND3*, *CCNE1*, *CDK2 *[p33], *CDK4*, *CDK6*, *CDKN1A *[p21, Cip1], *CDKN1B *[p27, Kip1], *CDKN2A *[p16], *CDKN2B *[p15], *CDKN2C *[p18], and *CDKN2D *[p19]) and survival among women diagnosed with invasive breast cancer participating in the SEARCH (Studies of Epidemiology and Risk factors in Cancer Heredity) breast cancer study. DNA from up to 4,470 women was genotyped for 85 polymorphisms that tag the known common polymorphisms (minor allele frequency > 0.05) in the genes. The genotypes of each polymorphism were tested for association with survival using Cox regression analysis.

**Results:**

The rare allele of the tagging single nucleotide polymorphism (SNP) rs2479717 is associated with an increased risk of death (hazard ratio = 1.26 per rare allele carried, 95% confidence interval: 1.12 to 1.42; *P *= 0.0001), which was not attenuated after adjusting for tumour stage, grade, and treatment. This SNP is part of a large linkage disequilibrium block, which contains *CCND3*, *BYSL*, *TRFP*, *USP49*, *C6ofr49*, *FRS3*, and *PGC*. We evaluated the association of survival and somatic expression of these genes in breast tumours using expression microarray data from seven published datasets. Elevated expression of the *C6orf49 *transcript was associated with breast cancer survival, adding biological interest to the finding.

**Conclusion:**

It is possible that *CCND3 *rs2479717, or another variant it tags, is associated with prognosis after a diagnosis of breast cancer. Further study is required to validate this finding.

## Introduction

Excluding non-melanoma skin cancer, breast cancer is the most common cancer in the UK, with 36,939 new cases diagnosed in 2004 [1]. The prognosis of breast cancer is generally good, with an overall 5-year survival rate of approximately 80% in England and Wales [2]. Clinical stage at diagnosis, including tumour size, lymph node status, and presence of metastases, along with tumour biological factors such as histological grade and type are the most important determinants of prognosis [3].

Cyclins and their regulators, which are involved in cell cycle control, are important as potential oncogenes or tumour suppressor genes in breast cancer [4]. The cell cycle consists of a series of well-controlled events that drive DNA replication and cell division. These events are divided into specific phases: preparation for DNA synthesis (G_1_), DNA synthesis (S), a gap phase (G_2_), and mitosis (M). Transition between these phases requires tight control; the G_1_/S phase transition, in particular, includes many cell cycle events that are altered in breast cancer [5]. Somatic alterations in these genes have been shown to correlate with breast cancer prognosis and survival [6-13], but few studies have examined the effects of inherited genetic variation in cell cycle genes. The *a*870*g *polymorphism of the *CCND1 *gene (rs603965) has been shown to be associated with breast cancer survival in a large Chinese population-based study [14] and in a small population of patients with metastatic breast cancer [15]. The V109G polymorphism of the p27 gene *CDKN1B *(rs2066827), examined by polymerase chain reaction analysis of tumour specimens, was associated with shortened disease-free survival in a subset of patients with infiltrating metastasis-free breast cancer [16].

These previous studies, however, were only of selected single nucleotide polymorphisms (SNPs), and the genes involved in the G_1 _phase of cell cycle control have not been systematically evaluated. The purpose of this study was to assess whether common germline genetic variation in these genes is associated with breast cancer survival by using a comprehensive SNP tagging approach to efficiently capture the common variation. Thirteen genes involved in the G_1 _phase of the cell cycle have been investigated in this study, including those that encode for the cyclin family that regulate cyclin-dependent kinases (*CCND1*, *CCND2*, *CCND3*, and *CCNE1*); cyclin-dependent kinases, which are necessary for the G_1_/S transition (*CDK2 *[p33], *CDK4*, and *CDK6*); and cyclin-dependent kinase inhibitors (*CDKN1A *[p21, Cip1], *CDKN1B *[p27, Kip1], *CDKN2A *[p16], *CDKN2B *[p15], *CDKN2C *[p18], and *CDKN2D *[p19]).

## Materials and methods

### Study population

Cases were selected from the Studies of Epidemiology and Risk factors in Cancer Heredity (SEARCH) breast cancer study, an ongoing population study of women diagnosed with breast cancer in the region of England included in the Eastern Cancer Registration and Information Centre (ECRIC) (formerly the East Anglian Cancer Registry). Eligible participants include women diagnosed with invasive breast cancer who were either under 70 years of age since the beginning of the study on 1 July 1996 (incident cases) or age 55 or younger since 1 January 1991 and who were alive at the start of the study (prevalent cases). Due to boundary changes, some prevalent cases diagnosed before 1995 were identified by the North Thames Cancer Registry.

Of those eligible, 67% returned a comprehensive epidemiological questionnaire and 64% returned a blood sample for genotyping. All participants in the study provided informed consent, and the study was approved by the Eastern Multicentre Research Ethics Committee. DNA is available from 4,470 cases for genotyping; 27% of these participants are prevalent cases.

The samples have been split into two sets in order to save DNA and reduce genotyping costs. Cases with high genomic yield were randomly selected from the first 3,500 recruited to comprise set 1 (n = 2,270), with set 2 comprising the remainder of these plus the next 970 incident cases recruited (n = 2,200). DNA yield was not associated with genotype for those cases included in set 1 or set 2. SNPs showing a positive association with survival after a diagnosis of breast cancer (*P *trend < 0.05) were genotyped in set 2. Data from both sets were then combined (n = 4,470) to jointly analyze the SNPs with positive associations. This joint analysis approach results in increased power to detect genetic association despite more stringent significance levels with Bonferroni correction [17].

As the prevalent cases were the first recruited, the proportion of prevalent cases was somewhat higher in set 1 than set 2 (33% versus 20%). In total, 1,370 prevalent cases were included in both sets; median time from diagnosis to blood draw was 3.4 years (range: 0.8 to 9.34 years). Median age at diagnosis was similar in the two sets (50 and 53 years old, respectively). Median time from diagnosis to blood draw was slightly longer for set 2 (18 months) than for set 1 (9 months), but the number of deaths in each set was similar (359 in set 1 and 278 in set 2). There was no significant difference in the morphology, histopathological grade, or TNM (tumour, node, metastasis) stage [18] of the cases by set or by prevalent/incident status.

### Participant follow-up

The ECRIC and the North Thames Cancer Registry have active follow-up at years 3 and 5 after diagnosis and then at 5-year intervals. Follow-up information and all-cause mortality are obtained by searching hospital information systems for recent visits. If a patient has not had a recent visit, the patient's general practitioner is contacted to obtain the vital status. Death certificate flagging through the Office of National Statistics also provides the registries with notification of deaths. The lag times with this process are a few weeks for cancer deaths and 2 months to a year for non-cancer deaths. Cause-specific mortality was obtained from part I of the death certificate.

### Gene/single nucleotide polymorphism selection

Thirteen genes involved in the G_1 _phase of cell cycle regulation were selected as candidate genes for breast cancer survival. A comprehensive SNP tagging approach was used in which tagging SNPs (tagSNPs) were chosen to capture all known common genetic variation in each gene with an estimated correlation coefficient (*r*^2^) of greater than 0.8. In some cases, SNPs that were poorly correlated with other single SNPs could be efficiently tagged with a haplotype defined by multiple SNPs. Correlation between these SNPs and the haplotype of tagSNPs (*r*^2^_s_) was aimed to be greater than 0.8. TagSNPs were identified with the program Tagger [19]. Data from the International HapMap Project [20] and resequencing data from the National Institute of Environmental Health Sciences (NIEHS) Environmental Genome Project [21] were used to select tagSNPs. In total, 85 tagSNPs were chosen.

### Genotyping

Genotyping was carried out using the TaqMan^® ^platform (Applied Biosystems, Foster City, CA, USA) according to the manufacturer's instructions. Primers and FAM- and VIC-labeled probes were supplied directly by Applied Biosystems as Assays-by-Design™. All assays were carried out in 384-well plates. Each plate included negative controls (with no DNA) and positive controls duplicated on a separate quality control plate. Plates were read on the ABI Prism 7900 using Sequence Detection Software (Applied Biosystems). Failed genotypes were not repeated. Assays in which the genotypes of duplicate samples did not show greater than 95% concordance were discarded and replaced with alternative assays with the same tagging properties. Call rates for each assay were over 95%.

### Statistical methods

Cox regression analysis was performed to determine the effect of each tagSNP on survival. The proportional hazards assumption was evaluated by visual inspection of log-log plots as well as tested analytically using Schoenfeld residuals. TagSNPs significantly associated with survival were re-evaluated in a model adjusted for known breast cancer prognostic factors, which included age at diagnosis (<40, 40 to 49, 50 to 59, or >60 years), clinical stage (TNM stage 1, 2, 3, or 4), histopathological grade (well differentiated, moderately differentiated, or poorly differentiated), estrogen receptor (ER) status, and treatment.

Time at risk began on the date of blood sample receipt and ended on the date of death from any cause or, if death did not occur, on 30 November 2006. This allows for the difference in ascertainment of incident and prevalent cases and provides an unbiased estimate of the relative hazard provided that the proportional hazards assumption is correct. Follow-up was censored at 10 years after diagnosis as follow-up became less reliable for each individual after 10 years. A hazard ratio (HR) was estimated for heterozygous and rare homozygous genotypes relative to the common genotype. Primary tests used were a likelihood ratio test (2 degrees of freedom) for heterogeneity of risk among the three genotypes (common homozygote, heterozygote, and rare homozygote) and a trend test (1 degree of freedom) based on the number of rare alleles carried. All analyses were performed with Intercooled Stata, version 8.2 (StataCorp LP, College Station, TX, USA).

## Results

### Survival analysis

The characteristics of the SEARCH breast cancer study participants for whom genotyping and vital status data were available are described in Table 1. More than 99% of the cases were Caucasian. There were 3,263 (73%) cases enrolled as incident cases and 1,207 (27%) as prevalent cases. No significant difference in survival hazard was found between the two groups (*P *= 0.19). During the 25,049 person-years at risk, there were 637 deaths before 10 years of follow-up. Five hundred forty-two of these deaths were coded as breast cancer-specific.

**Table 1:** SEARCH participant survival characteristics

	Set 1		Sets 1 and 2	
	n = 2,270		n = 4,470	
Total time at risk, years	1,3851.47		2,5049.24	
Median follow-up, years^a^	7.75	(0.56–10)^b^	7.45	(0.48–10)^b^
Median time at risk, years	6.47	(0.10–9.64)^b^	5.70	(0.03–9.77)^b^
Median time from diagnosis to study entry, years	0.73	(0.00–8.64)^b^	1.16	(0.00–9.34)^b^
Number of deaths	359		637	
Annual mortality rate	0.026		0.025	
5-year survival rate	0.88	(0.86–0.89)^c^	0.88	(0.87–0.89)^c^
Median age at diagnosis, years	50.2	(25–69)^b^	51.0	(23–69)^b^
Age at diagnosis, years				
<40	212	9.30%	394	8.81%
40–49	753	33.20%	1,331	29.78%
50–59	997	43.90%	1,802	40.31%
60+	308	13.60%	943	21.10%
Histopathological grade				
Well differentiated	434	19.11%	871	19.49%
Moderately differentiated	787	34.67%	1,687	37.74%
Poorly differentiated	504	22.20%	1,019	22.80%
Unknown	545	24.00%	893	19.98%
Morphological type				
Ductal	1,674	73.74%	3,316	74.18%
Lobular	351	15.46%	659	14.74%
Other	222	9.78%	455	10.17%
Unknown	23	1.01%	40	0.89%
Clinical stage				
1	1,112	48.99%	2,190	48.99%
2	986	43.33%	1,981	44.32%
3 or 4	172	7.58%	194	4.34%
Missing	0	0%	105	2.35%

^a^Follow-up censored at 10 years. ^b^Range of variable. ^c^95% confidence interval. SEARCH, Studies of Epidemiology and Risk factors in Cancer Heredity.

#### Set 1 analysis

The results of the univariate Cox regression analyses for single marker tagSNPs are shown in Supplementary Table 1 and for multimarker tagSNPs in Supplementary Table 2 (Additional File 1). None of the tagSNPs in *CCND1*, *CCND2*, *CCNE1*, *CDK2*, *CDK4*, *CDK1A*, *CDKN1B*, *CDKN2A*, *CDKN2B*, *CDKN2C*, or *CDKN2D *were significantly associated with all-cause survival. The trend tests for *CCND3 *rs2479717, *CCND3 *rs9529, and *CDK6 *rs2079147 were significant at the 0.05 level (*P *= 0.001, 0.006, and 0.02, respectively). *CCND3 *rs2479717 and *CCND3 *rs9529 are highly correlated with each other (*r*^2 ^= 0.92). In a forced Cox regression model including both *CCND3 *rs2479717 and *CCND3 *rs9529, only *CCND3 *rs2479717 remained significant (*P *= 0.029). Therefore, only *CCND3 *rs2479717 and *CDK6 *rs2079147 were genotyped in the second set.

#### Joint analysis

**Figure 1:**
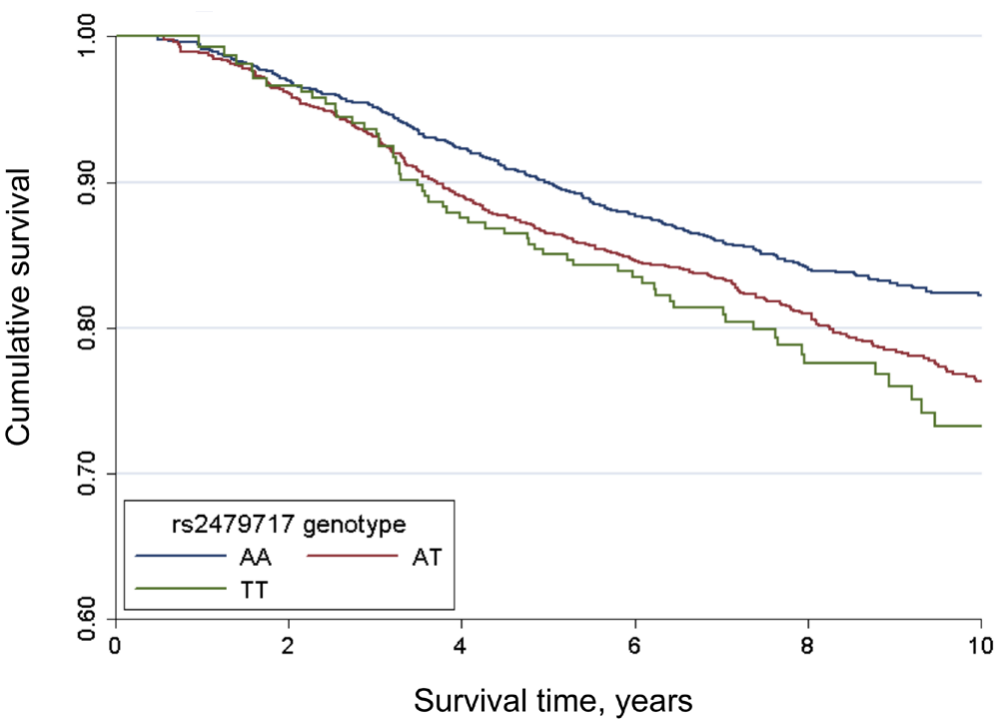
Kaplan-Meier survival function by *CCND3 *rs2479717 genotype. Kaplan-Meier survival probabilities for women diagnosed with invasive breast cancer in the SEARCH (Studies of Epidemiology and Risk factors in Cancer Heredity) breast cancer study by rs2479717 genotype are shown.

*CCND3 *rs2479717 remained significant in the joint analysis (HR per rare allele carried = 1.26, 95% confidence interval [CI]: 1.12 to 1.42; *P *= 0.0001) (Figure 1). Data on tumour grade, TNM stage, and age at diagnosis were available for 80%, 97%, and 100% of the cases, respectively. Only stage and grade remained significantly associated with survival in the multivariate model. Radiotherapy, chemotherapy, and adjuvant hormone therapy treatment data were available for 4,303 (96.3%) cases. Of these, 1,412 (32.8%) underwent chemotherapy, 3,006 (69.9%) were treated with adjuvant hormone therapy, and 3,099 (72.0%) received radiotherapy. Surgical treatment information was available for 4,194 (93.8%) cases; of these, 3,840 (91.6%) underwent surgery. The risk associated with *CCND3 *rs2479717 was not significantly attenuated after adjusting for tumour stage, grade, radiotherapy, chemotherapy, adjuvant hormone therapy, and surgery (HR per rare allele carried = 1.23, 95% CI: 1.07 to 1.41; *P *= 0.003). We repeated the analysis in those recruited within 3 years of diagnosis (3,558 individuals), and there were no differences in the results for both the univariate analysis (HR per rare allele carried = 1.28, 95% CI: 1.13 to 1.46; *P *= 0.0002) and the multivariate model (HR per rare allele carried = 1.23, 95% CI: 1.07 to 1.42; *P *= 0.004).

ER status was available for 2,624 (58.7%) cases. Of these, 1,975 (75.3%) individuals had an ER-positive tumour. No differences across stage, grade, or ER status are noted across the rs2479717 genotype (Table 2). If ER status is added to the multivariate model, the power of the model is somewhat decreased due to a reduction in sample size, resulting in a slight attenuation of the HR and loss of significance for *CCND3 *rs2479717 (HR = 1.13, 95% CI: 0.94 to 1.35; *P *= 0.19). The HR for *CCND3 *rs2479717 was almost the same for those individuals with an ER-positive tumour (HR per rare allele carried = 1.13; 95% CI: 0.91 to 1.41) and those with an ER-negative tumour (HR per rare allele carried = 1.12, 95% CI: 0.89 to 1.43); the test for interaction was not significant (*P *= 0.97).

**Table 2:** *CCND3 *rs2479717 genotype frequency across stage, grade, and estrogen receptor status

	*CCND3 *rs2479717 genotype			
	AA	AT	TT	Missing
Stage				
1	1,193 (54.5%)	808 (36.9%)	142 (6.5%)	48 (2.2%)
2	1,031 (52.0%)	753 (38.0%)	145 (7.3%)	54 (2.7%)
3 or 4	90 (46.4%)	82 (42.3%)	17 (8.8%)	5 (2.6%)
Missing	60 (58.8%)	31 (30.4%)	9 (8.8%)	2 (2.0%)
Grade				
1	484 (55.4%)	314 (35.9%)	60 (6.9%)	16 (1.8%)
2	889 (52.7%)	658 (39.0%)	104 (6.2%)	36 (2.1%)
3	533 (52.3%)	375 (36.8%)	86 (8.4%)	26 (2.5%)
Missing	468 (52.5%)	327 (36.7%)	63 (7.1%)	34 (3.8%)
Estrogen receptor status				
Positive	1,089 (55.1%)	717 (36.3%)	130 (6.6%)	39 (2.0%)
Negative	320 (49.3%)	262 (40.4%)	53 (8.2%)	14 (2.2%)
Missing	965 (52.3%)	695 (37.6%)	130 (7.0%)	56 (3.0%)

Results were consistent for breast cancer-specific mortality (Supplementary Tables 3 and 4 found in Additional File 2). Only *CCND3 *rs2479717 remained significant in the joint analysis (HR per rare allele carried = 1.26, 95% CI: 1.11 to 1.44; *P *= 0.0004). This HR was not attenuated by stage, grade, and treatment (HR per rare allele carried = 1.21, 95% CI: 1.04 to 1.40; *P *= 0.01).

### Somatic expression analysis

**Figure 2:**
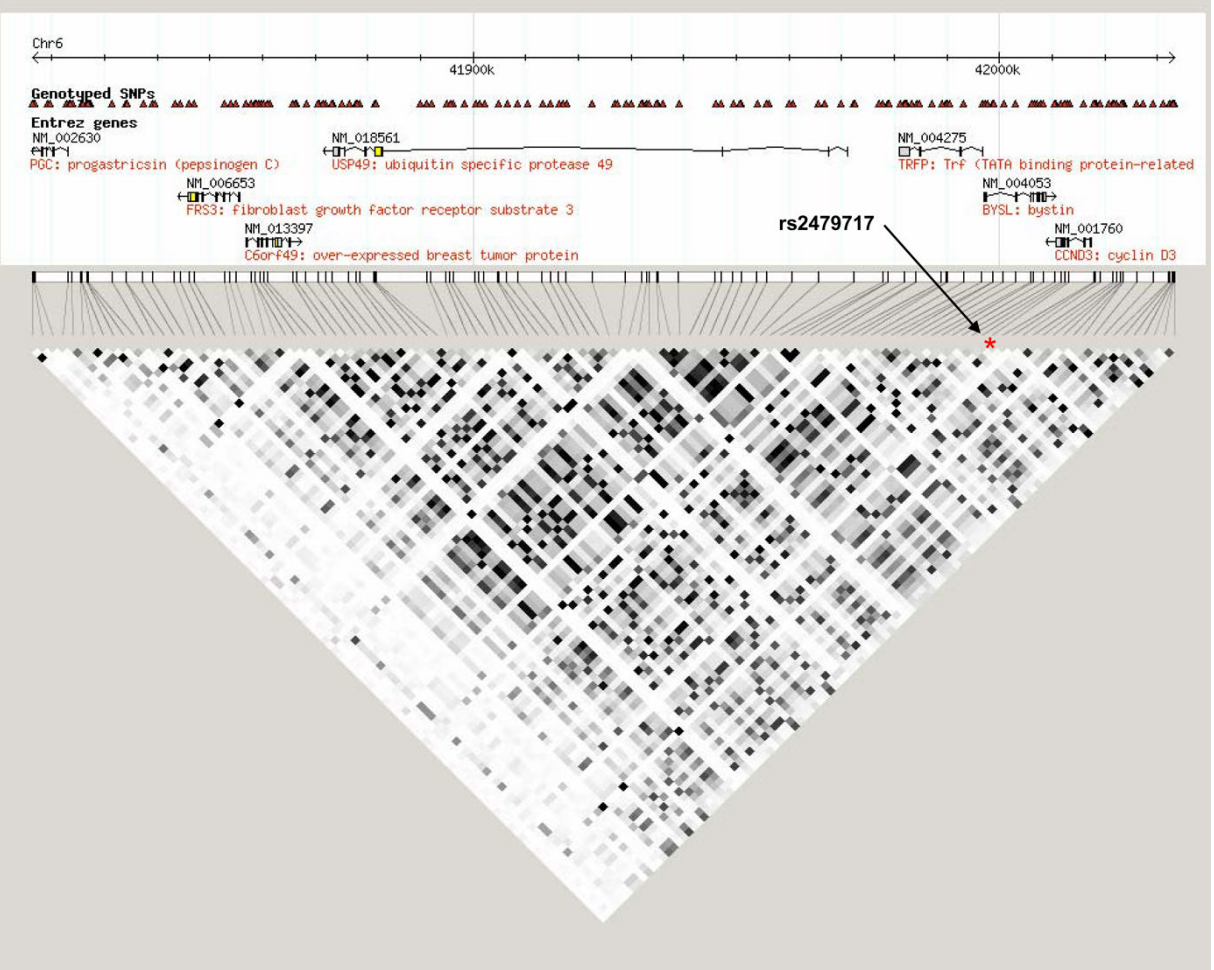
Linkage disequilibrium block surrounding *CCND3 *rs2479717. This 219-kilo-base pair section on chromosome 6 contains the genes *CCND3*, *BYSL*, *TRFP*, *USP49*, *C6ofr49*, *FRS3*, and *PGC*, mapped relative to common single nucleotide polymorphisms on HapMap CEU. Squares indicate pairwise *r*^2 ^on a grayscale (black = 1, white = 0). The position of rs2479717 is denoted by a red asterisk.

*CCND3 *rs2479717 is an a > t intronic alteration approximately 50 bases downstream from exon 3. It lies in a large linkage disequilibrium (LD) block on chromosome 6, which contains seven genes: *CCND3*, *BYSL*, *TRFP*, *USP49*, *C6ofr49*, *FRS3*, and *PGC *(Figure 2). To further evaluate the effect of these genes on breast cancer survival, microarray gene expression data for seven breast cancer cohorts were taken from existing literature and public databases (Gene Expression Omnibus and ArrayExpress) [22-28]. The publicly available data comprise raw expression data that have been 'normalized'. This usually involves background correction, quantile normalization, and log transformation. The retrieved datasets were further normalized, if necessary, by transforming them onto a common log2 scale and shifting the median of each array to zero [29].

Study characteristics are included in Table 3. In total, the studies included tumour samples from 1,241 individuals. All of the studies contained clinical outcomes; five of the studies had information on all-cause mortality, and two of the studies had disease recurrence as an outcome. Most of the studies had a mix of untreated and treated individuals, with treatment including both chemotherapy and/or hormonal therapy. Median age at diagnosis ranged from less than 53 years to 65 years. The majority of tumours were ER-positive and medium grade (Supplementary Table 5 found in Additional File 3). Combined, the datasets provided 9,048 years at risk (median of 7.4 years at risk) and a total of 384 events.

**Table 3:** Microarray study characteristics

Study	Institution	Outcome	Microarray platform	Evaluated genes	Number of samples^a^	Total time at risk, years	Median time at risk, years	Number deaths/recurrences
Blenkiron, *et al*. [22]	Nottingham City Hospital (Nottingham, UK)	All-cause mortality	Illumina (San Diego, CA, USA)	*PGC*, *FRS3*, *C6orf49*, *TRFP*, *BYSL*, *USP49*, *CCND3*	128	1,198.0	11.1	45
Chin, *et al*. [23]	University of California at San Francisco	All-cause mortality	Affymetrix U133A (Santa Clara, CA, USA)	*PGC*, *FRS3*, *C6orf49*, *TRFP*, *BYSL*, *CCND3*	129	822.6	6.0	45
Miller, *et al*. [24]	Uppsala, Sweden	All-cause mortality	Affymetrix U133A	*PGC*, *FRS3*, *C6orf49*, *BYSL*, *CCND3*	234	1,927.5	10.2	54
Sørlie, *et al*. [25]	Stanford University (Stanford, CA, USA)	All-cause mortality	cDNA	*BYSL*, *USP49*, *CCND3*	76	227.6	2.5	30
Sotiriou, *et al*. [26]	John Radcliffe (Oxford, UK)	Disease recurrence	Affymetrix U133A	*PGC*, *FRS3*, *TRFP*, *BYSL*, *CCND3*	94	709.9	7.3	24^b^
van de Vijver, *et al*. [27]	The Netherlands Cancer Institute (Amsterdam)	All-cause mortality	Agilent Technologies, Inc. (Santa Clara, CA, USA)	*PGC*, *FRS3*, *C6orf49*, *BYSL*, *USP49*, *CCND3*	295	2,319.8	7.2	79
Wang, *et al*. [28]	Erasmus (Rotterdam, The Netherlands)	Disease recurrence	Affymetrix U133A	*PGC*, *C6orf49*, *FRS3*, *USP49*, *BYSL*, *CCND3*	285	1,843.1	7.2	107^b^
Total					1,241	9,048.4	7.4	384

^a^With endpoint data. ^b^Disease recurrence.

**Figure 3:**
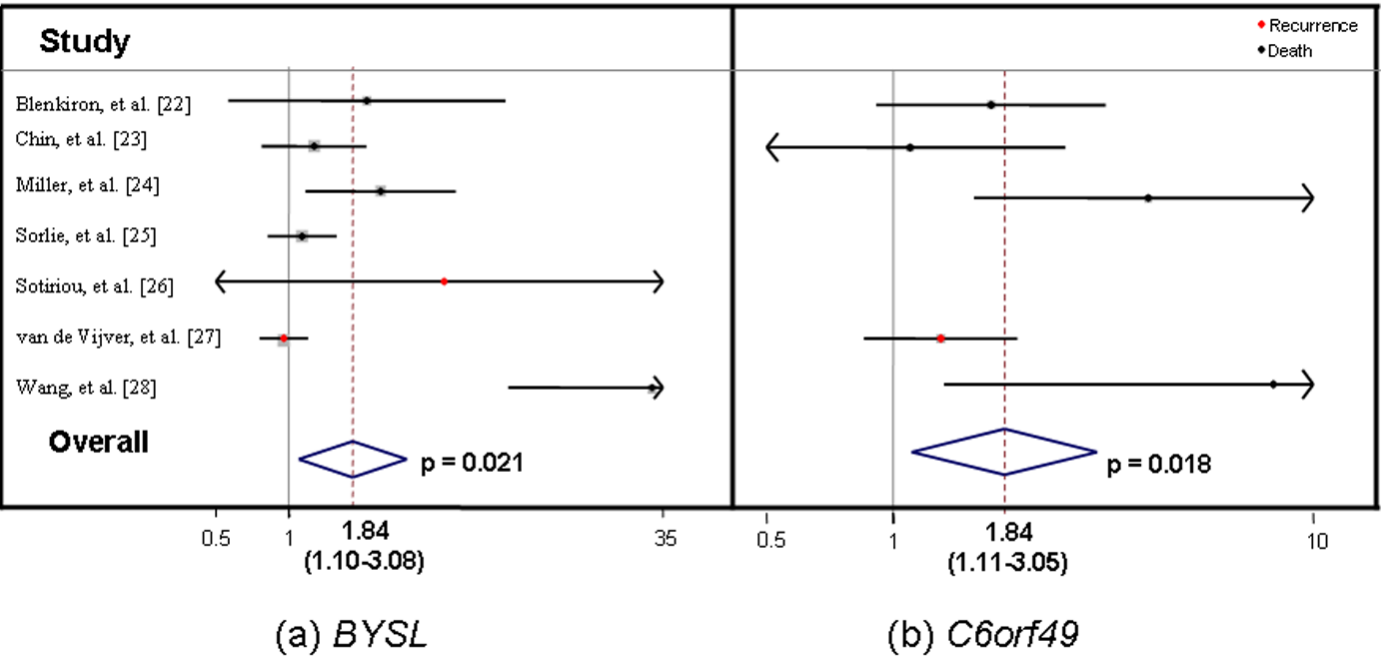
Association of *BYSL ***(a) **and *C6orf49 ***(b) **breast tumour expression with breast cancer outcome. Forest plots are shown for random-effects meta-analyses of breast tumour expression of *BYSL *and *C6orf49 *evaluated by microarray and association with breast cancer recurrence or death. Study-specific hazard ratios with confidence intervals and an overall estimate of effect are shown. Grey boxes indicate the weight of study in random-effects meta-analysis.

To test whether a gene's expression is associated with clinical outcome, we used Cox proportional hazards regression models. In this context, the HR refers to the proportional increase in hazard risk per unit increase on a log2 scale of expression level of the transcript. To attempt to control for study heterogeneity, expression data for each gene were analyzed two ways: a Cox survival model stratified by study (fixed effects) and a random-effects meta-analysis. Elevated expression levels of the *BYSL *transcript were significantly associated with breast cancer survival in a random-effects model (HR = 1.84, 95% CI: 1.10 to 3.08; *P *= 0.02) (Figure 3a), but the association did not reach significance in a fixed-effects model (HR = 1.17, 95% CI: 0.99 to 1.38; *P *= 0.08). Elevated expression levels of the *C6orf49 *transcript were significantly associated with breast cancer survival in a fixed-effects model (HR = 1.60, 95% CI: 1.18 to 2.16; *P *= 0.002) and in a random-effects model (HR = 1.84, 95% CI: 1.11 to 3.05; *P *= 0.02) (Figure 3b). Expression levels of *CCND3*, *TRFP*, *USP49*, *FRS3*, or *PGC *transcripts were not significantly associated with breast cancer survival (Supplementary Table 6 found in Additional File 3).

## Discussion

We have evaluated the association of 85 tagSNPs in 13 cell cycle control genes with survival after a diagnosis of breast cancer. Previous work has shown that expression of these genes is associated with breast cancer prognosis; however, to our knowledge, this is the first study to systematically assess germline variation in genes involved in controlling the cell cycle and breast cancer survival.

This study used a two-stage design, with an initial set of 85 tagSNPs genotyped in 2,270 individuals. The top two SNPs, with a *P *value of less than 0.05, were genotyped in the second set of patients (n = 2,200). Because a combined analysis with adjustment for multiple testing has been shown to increase power over a replication study, a joint analysis of both sets of data was performed. One SNP, *CCND3 *rs2479717, showed a significant association with survival after a diagnosis of breast cancer (unadjusted *P *value = 0.0001). This finding remains significant after a conservative Bonferroni correction for multiple testing (*P *value = 0.0085), and the HR is not significantly attenuated after adjusting for stage, grade, and treatment. There was no evidence of association with survival for polymorphisms in *CCND1*, *CCND2*, *CCNE1*, *CDK2*, *CDK4*, *CDK6*, *CDKN1A*, *CDKN1B*, *CDKN2A*, *CDKN2B*, *CDKN2C*, and *CDKN2D*.

These findings were based on the analysis of all-cause mortality. This may result in a reduction of statistical power as some deaths will be unrelated to breast cancer. Breast cancer-specific mortality was available from death certificates, and the results were consistent for the breast cancer-specific analysis, with an identical HR for *CCND3 *rs2479717. It is worth noting that cause of death as coded on a death certificate is also prone to misclassification and subsequent loss of statistical power.

Our analyses incorporated prevalent cases. It may be thought that inclusion of prevalent cases may result in a bias of the HR. However, provided that the Cox proportional hazards assumption holds true, the HR estimate is unbiased. For example, there is no significant difference between the HRs for *CCND3 *rs2479717, TNM stage, or histopathological grade when comparing subjects recruited within 6 months of diagnosis with those recruited more than 6 months after diagnosis (*P *= 0.69, 0.90, and 0.42, respectively). Furthermore, our repeated analyses, including only those individuals recruited within 3 years of their diagnosis, were identical to our full analysis.

*CCND3 *encodes for cyclin D3, a protein involved in the regulation of the G_1_/S phase transition. The SNP associated with survival, *CCND3 *rs2479717, is in an intron and unlikely to have a functional effect. However, a functional effect would not be expected as it was chosen as a tagSNP, not as a functional SNP. Furthermore, a functional variant tagged by this SNP may not even alter the function of *CCND3*; the SNP lies in a large LD block with several genes that are reasonable candidates for breast cancer survival. *PGC *encodes for pepsinogen C, a proteolytic enzyme involved in digestion, which is expressed in breast tumours [30]. Higher pepsinogen C expression is associated with well-differentiated and moderately differentiated breast tumours [31] and has been associated with longer overall survival in these patients [32,33]. *BYSL *encodes for bystin, a crucial component protein of an adhesion molecule complex that is important for the attachment of the embryo to the uterus [34]. This protein is present in human prostatic carcinoma cells in areas of perineural invasion in an increasing gradient, suggesting a role in perineural adhesion [35]. *C6orf49 *encodes for overexpressed breast tumour protein, a member of the LIM domain (cysteine-rich double zinc fingers) protein family that is overexpressed in tumours and has a possible role in cancer differentiation [36,37]. *FRS3 *encodes for fibroblast growth factor receptor substrate 3, a negative regulator in epidermal growth factor receptor tyrosine kinase signaling pathways [38,39]. *USP49 *encodes for ubiquitin-specific protease 49, which is involved in the modification of cellular proteins. Ubiquitin-specific protease 49 is expressed in samples derived from tumour biopsies [40]. *TRFP *encodes for TATA-binding protein-related protein, which is associated with an RNA polymerase II-SRB complex; this complex may regulate class II genes [41].

To further evaluate this LD block, we examined breast tumour expression of these seven genes using expression microarray data from seven published datasets. Significant associations between increased tumour expression levels of *BYSL *and *C6orf49 *transcripts and breast cancer survival emerged. Differences between the microarray datasets, varying outcome information, and incomplete control of confounding by prognostic factors may limit interpretation of these findings; however, we attempted to control for patient and tumour heterogeneity between these studies by performing two analyses: a random-effects and a fixed-effects meta-analysis. Although the results for *BYSL *are unclear, both analyses showed consistent associations of elevated tumour expression of the *C6orf49 *transcript with survival after a diagnosis of breast cancer.

## Conclusion

Our study has found evidence that tagSNP *CCND3 *rs2479717, which is found in a genomic region that includes *CCND3 *and six other genes, is associated with survival after a diagnosis of breast cancer. Although our study began as an evaluation of cell cycle control genes, our significant finding may actually relate to a gene in LD with *CCND3 *rs2479717 that is not related to cell cycle control. This is supported by our findings that elevated tumour expression of the *C6orf49 *transcript, one of the genes in LD with rs2479717, is associated with breast cancer survival. If our findings can be confirmed in other studies, further evaluation of this locus to identify the causal variant would be warranted.

## Abbreviations

CI = confidence interval; ECRIC = Eastern Cancer Registration and Information Centre; ER = estrogen receptor; HR = hazard ratio; LD = linkage disequilibrium; SEARCH = Studies of Epidemiology and Risk factors in Cancer Heredity; SNP = single nucleotide polymorphism; tagSNP = tagging single nucleotide polymorphism; TNM = tumour, node, metastasis.

## Competing interests

The authors declare that they have no competing interests.

## Authors' contributions

EMA carried out statistical analyses and drafted the manuscript. KED carried out genotyping, ER immunohistochemistry, and data cleaning. FL carried out genotyping and selected SNPs for evaluation. MS is responsible for patient recruitment. DG is responsible for collecting patient characteristics and patient follow-up data. DFE is a co-investigator in SEARCH and is involved in study design. AET was responsible for microarray data retrieval and cleaning and advised on data analysis. CC and NEC contributed to study design and interpretation of results. PDPP is a co-investigator in SEARCH, conceived of the study, participated in its design and coordination, and helped to draft the manuscript. All authors read and approved the final manuscript.

## Supplementary Material

Additional File 1 This file contains Supplementary tables 1 and 2, which show the results of the univariate all cause mortality Cox regression analyses for single marker tagSNPs and multimarker tagSNPs. Click here for file

Additional File 2 This file contains Supplementary tables 3 and 4, which show the results of the univariate breast cancer specific mortality Cox regression analyses for single marker tagSNPs and multimarker tagSNPs. Click here for file

Additional File 3 This file contains Supplementary tables 5 and 6, which show additional information regarding the microarray datasets used in the somatic expression analyses. Supplementary table 5 shows additional microarray study and patient characteristics. Supplementary table 6 shows hazard ratios associated with microarray expression of genes in linkage disequilibrium with *CCND3 *rs2479717. Click here for file
