# Supplementary material for: Effects of common germline genetic variation in cell cycle control genes on breast cancer survival: results from a population-based cohort
Source: Breast Cancer Res. 2008 May 28;10(3):R47. doi: 10.1186/bcr2100 (PMC2481496; doi:10.1186/bcr2100)
Supplement: Additional File 1 — This file contains Supplementary tables 1 and 2, which show the results of the univariate all cause mortality Cox regression analyses for single marker tagSNPs and multimarker tagSNPs. [file bcr2100-S1.doc]

**Supplementary table 1. All-cause mortality hazard ratios associated with common tagSNPs in cell cycle genes after a diagnosis of breast c**ancer

|  | **dbSNP** | **Genotype frequencies** | | | | **Trend test** | | **Risk per allele** | | | **Heterogeneity test** | | **Heterozygote Risk** | | | **Homozygote Risk** | | |
| --- | --- | --- | --- | --- | --- | --- | --- | --- | --- | --- | --- | --- | --- | --- | --- | --- | --- | --- |
| **Gene** | **Reference** | **AA** | **Aa** | **aa** | *Total* | **x^2^** | **P-value** | **HR** | **LCL** | **UCL** | **x^2^** | **P-value** | **HR** | **LCL** | **UCL** | **HR** | **LCL** | **UCL** |
| *CCND1* | rs7178 | 1744 | 282 | 12 | 2038 | 0.071 | 0.790 | 1.039 | 0.785 | 1.375 | 1.049 | 0.592 | 1.096 | 0.813 | 1.477 | 0.487 | 0.068 | 3.471 |
|  | rs3862792 | 1917 | 122 | 1 | 2040 | 1.259 | 0.262 | 1.270 | 0.849 | 1.901 | 2.610 | 0.271 | 1.190 | 0.779 | 1.818 | 6.367 | 0.893 | 45.397 |
|  | rs603965 | 627 | 982 | 428 | 2037 | 0.000 | 0.991 | 1.001 | 0.861 | 1.164 | 0.013 | 0.993 | 0.989 | 0.772 | 1.268 | 1.004 | 0.742 | 1.359 |
|  | rs678653 | 811 | 946 | 263 | 2020 | 0.058 | 0.809 | 0.980 | 0.834 | 1.152 | 1.330 | 0.514 | 0.889 | 0.704 | 1.123 | 1.036 | 0.740 | 1.451 |
|  | rs602652 | 612 | 1057 | 502 | 2171 | 0.144 | 0.705 | 0.972 | 0.837 | 1.128 | 0.617 | 0.734 | 1.042 | 0.811 | 1.339 | 0.935 | 0.689 | 1.268 |
|  | rs3212879 | 614 | 1076 | 483 | 2173 | 0.006 | 0.939 | 1.006 | 0.866 | 1.169 | 0.172 | 0.917 | 0.965 | 0.751 | 1.239 | 1.017 | 0.755 | 1.370 |
|  | rs3212891 | 692 | 1067 | 431 | 2190 | 0.000 | 0.998 | 1.000 | 0.860 | 1.163 | 0.362 | 0.834 | 0.943 | 0.741 | 1.201 | 1.015 | 0.752 | 1.370 |
| *CCND2* | rs3217795 | 1756 | 409 | 21 | 2186 | 0.001 | 0.976 | 1.004 | 0.785 | 1.284 | 1.002 | 0.606 | 0.944 | 0.717 | 1.244 | 1.538 | 0.635 | 3.724 |
|  | rs3217805 | 812 | 1021 | 330 | 2163 | 0.002 | 0.963 | 0.996 | 0.855 | 1.161 | 2.427 | 0.297 | 1.142 | 0.906 | 1.439 | 0.913 | 0.650 | 1.281 |
|  | rs3217820 | 842 | 1056 | 283 | 2181 | 0.081 | 0.776 | 1.023 | 0.874 | 1.197 | 2.603 | 0.272 | 0.891 | 0.708 | 1.122 | 1.143 | 0.832 | 1.570 |
|  | rs3217869 | 808 | 1050 | 325 | 2183 | 0.279 | 0.598 | 0.959 | 0.821 | 1.120 | 4.713 | 0.095 | 0.796 | 0.632 | 1.003 | 1.025 | 0.754 | 1.395 |
|  | rs3217926 | 794 | 1061 | 330 | 2185 | 0.283 | 0.595 | 1.043 | 0.894 | 1.217 | 2.791 | 0.248 | 0.903 | 0.714 | 1.141 | 1.162 | 0.856 | 1.577 |
|  | rs3217936 | 1004 | 936 | 247 | 2187 | 0.097 | 0.756 | 1.025 | 0.876 | 1.200 | 2.257 | 0.324 | 0.905 | 0.720 | 1.138 | 1.166 | 0.840 | 1.619 |
|  | rs3217852 | 1283 | 778 | 128 | 2189 | 0.004 | 0.952 | 0.995 | 0.836 | 1.184 | 0.028 | 0.986 | 1.006 | 0.803 | 1.260 | 0.966 | 0.610 | 1.530 |
|  | rs3217862 | 1511 | 605 | 71 | 2187 | 0.430 | 0.512 | 0.935 | 0.764 | 1.144 | 8.432 | 0.015 | 0.757 | 0.587 | 0.976 | 1.546 | 0.946 | 2.527 |
|  | rs3217863 | 1874 | 310 | 9 | 2193 | 0.438 | 0.508 | 0.904 | 0.668 | 1.223 | 3.014 | 0.222 | 0.821 | 0.590 | 1.141 | 2.219 | 0.711 | 6.921 |
|  | rs3217906 | 1214 | 814 | 157 | 2185 | 0.270 | 0.603 | 1.046 | 0.884 | 1.238 | 12.295 | 0.002 | 0.786 | 0.621 | 0.995 | 1.549 | 1.095 | 2.191 |
|  | rs3217916 | 1144 | 879 | 165 | 2188 | 0.010 | 0.921 | 0.992 | 0.838 | 1.173 | 3.775 | 0.151 | 0.853 | 0.680 | 1.070 | 1.212 | 0.837 | 1.756 |
|  | rs3217925 | 1213 | 825 | 133 | 2171 | 0.898 | 0.343 | 0.918 | 0.769 | 1.097 | 1.922 | 0.383 | 0.854 | 0.680 | 1.071 | 0.981 | 0.631 | 1.524 |
|  | rs3217933 | 1226 | 826 | 136 | 2188 | 0.045 | 0.832 | 0.981 | 0.825 | 1.168 | 0.191 | 0.909 | 0.955 | 0.763 | 1.195 | 1.018 | 0.655 | 1.581 |
|  | rs3217901 | 745 | 1074 | 371 | 2190 | 0.932 | 0.334 | 1.077 | 0.926 | 1.253 | 0.948 | 0.622 | 1.065 | 0.838 | 1.352 | 1.165 | 0.857 | 1.584 |
| *CCND3* | rs1410492 | 1191 | 847 | 146 | 2184 | 3.461 | 0.063 | 0.846 | 0.708 | 1.012 | 4.176 | 0.124 | 0.896 | 0.718 | 1.118 | 0.614 | 0.364 | 1.038 |
|  | rs2479717 | 2374 | 1674 | 313 | 4361 | 14.168 | **0.0001** | 1.264 | 1.121 | 1.425 | 14.563 | 0.001 | 1.273 | 1.077 | 1.505 | 1.433 | 1.075 | 1.909 |
|  | rs1051130 | 715 | 1015 | 458 | 2188 | 3.659 | 0.056 | 1.152 | 0.997 | 1.331 | 5.278 | 0.071 | 1.314 | 1.022 | 1.690 | 1.312 | 0.973 | 1.769 |
|  | rs3218092 | 1498 | 636 | 57 | 2191 | 0.972 | 0.324 | 0.903 | 0.735 | 1.109 | 1.565 | 0.457 | 0.859 | 0.674 | 1.095 | 0.999 | 0.531 | 1.880 |
|  | rs9529 | 1173 | 849 | 161 | 2183 | 7.646 | **0.006** | 1.257 | 1.072 | 1.475 | 8.755 | 0.013 | 1.365 | 1.094 | 1.705 | 1.429 | 0.972 | 2.100 |
|  | rs3218110 | 1216 | 825 | 142 | 2183 | 0.045 | 0.832 | 1.019 | 0.859 | 1.207 | 5.032 | 0.081 | 1.191 | 0.957 | 1.482 | 0.729 | 0.437 | 1.215 |
|  | rs3218114 | 1490 | 641 | 53 | 2184 | 0.742 | 0.389 | 0.914 | 0.743 | 1.124 | 0.767 | 0.681 | 0.905 | 0.713 | 1.149 | 0.876 | 0.433 | 1.772 |
| *CCNE1* | rs997669 | 761 | 1061 | 361 | 2183 | 1.581 | 0.209 | 1.103 | 0.947 | 1.284 | 1.990 | 0.370 | 1.038 | 0.817 | 1.318 | 1.240 | 0.915 | 1.682 |
|  | rs3218038 | 2027 | 157 | 1 | 2185 | . | . | . | . | . | . | . | . | . | . | . | . | . |
|  | rs3218076 | 1170 | 880 | 137 | 2187 | 0.069 | 0.793 | 0.977 | 0.821 | 1.162 | 0.072 | 0.965 | 0.981 | 0.786 | 1.223 | 0.947 | 0.603 | 1.488 |
|  | rs3218036 | 987 | 956 | 243 | 2186 | 1.142 | 0.285 | 1.090 | 0.931 | 1.276 | 2.375 | 0.305 | 0.992 | 0.790 | 1.246 | 1.281 | 0.921 | 1.782 |

|  | **dbSNP** | **Genotype frequencies** | | | | **Trend test** | | **Risk per allele** | | | **Heterogeneity test** | | **Heterozygote Risk** | | | **Homozygote Risk** | | |
| --- | --- | --- | --- | --- | --- | --- | --- | --- | --- | --- | --- | --- | --- | --- | --- | --- | --- | --- |
| **Gene** | **Reference** | **AA** | **Aa** | **aa** | *Total* | **x^2^** | **P-value** | **HR** | **LCL** | **UCL** | **x^2^** | **P-value** | **HR** | **LCL** | **UCL** | **HR** | **LCL** | **UCL** |
| *CDK2* | rs2069408 | 971 | 964 | 239 | 2174 | 0.037 | 0.847 | 1.016 | 0.868 | 1.189 | 0.460 | 0.795 | 1.071 | 0.856 | 1.340 | 0.979 | 0.683 | 1.403 |
|  | rs1045435 | 1838 | 326 | 20 | 2184 | 0.788 | 0.375 | 1.125 | 0.871 | 1.453 | 0.950 | 0.622 | 1.155 | 0.869 | 1.535 | 1.038 | 0.333 | 3.239 |
| *CDK4* | rs2270777 | 630 | 1028 | 383 | 2041 | 0.034 | 0.854 | 0.985 | 0.843 | 1.151 | 0.637 | 0.727 | 0.914 | 0.715 | 1.167 | 0.993 | 0.729 | 1.354 |
| *CDK6* | rs3731343 | 619 | 1083 | 466 | 2168 | 0.366 | 0.545 | 0.955 | 0.821 | 1.110 | 1.785 | 0.410 | 1.074 | 0.838 | 1.377 | 0.890 | 0.650 | 1.218 |
|  | rs3757823 | 1751 | 410 | 18 | 2179 | 0.163 | 0.687 | 1.052 | 0.823 | 1.346 | 1.438 | 0.487 | 0.984 | 0.748 | 1.294 | 1.797 | 0.742 | 4.352 |
|  | rs2079147 | 1227 | 2126 | 996 | 4349 | 2.895 | 0.089 | 1.101 | 0.986 | 1.229 | 3.349 | 0.187 | 1.043 | 0.861 | 1.263 | 1.212 | 0.975 | 1.508 |
|  | rs4729049 | 1803 | 364 | 14 | 2181 | 1.386 | 0.239 | 1.165 | 0.908 | 1.496 | 1.765 | 0.414 | 1.203 | 0.919 | 1.573 | 0.934 | 0.232 | 3.755 |
|  | rs8 | 1349 | 700 | 88 | 2137 | 0.082 | 0.774 | 1.028 | 0.852 | 1.240 | 2.133 | 0.344 | 0.929 | 0.735 | 1.174 | 1.366 | 0.844 | 2.210 |
|  | rs445 | 1735 | 412 | 24 | 2171 | 0.062 | 0.803 | 1.032 | 0.809 | 1.316 | 0.718 | 0.698 | 0.982 | 0.747 | 1.289 | 1.489 | 0.615 | 3.606 |
|  | rs992519 | 1614 | 512 | 47 | 2173 | 1.352 | 0.245 | 1.132 | 0.921 | 1.392 | 1.885 | 0.390 | 1.186 | 0.932 | 1.509 | 1.032 | 0.487 | 2.187 |
|  | rs42046 | 1141 | 874 | 152 | 2167 | 1.498 | 0.221 | 1.110 | 0.940 | 1.312 | 2.072 | 0.355 | 1.175 | 0.941 | 1.466 | 1.126 | 0.740 | 1.714 |
|  | rs8179 | 1325 | 724 | 96 | 2145 | 0.325 | 0.569 | 1.055 | 0.879 | 1.265 | 3.957 | 0.138 | 1.198 | 0.957 | 1.499 | 0.749 | 0.408 | 1.374 |
|  | rs2282991 | 1833 | 332 | 13 | 2178 | 0.104 | 0.747 | 1.046 | 0.796 | 1.374 | 0.157 | 0.924 | 1.034 | 0.773 | 1.383 | 1.279 | 0.318 | 5.137 |
|  | rs3731348 | 1914 | 252 | 3 | 2169 | . | . | . | . | . | . | . | . | . | . | . | . | . |
|  | rs2285332 | 1245 | 783 | 151 | 2179 | 0.287 | 0.592 | 0.954 | 0.804 | 1.133 | 0.294 | 0.863 | 0.949 | 0.756 | 1.190 | 0.921 | 0.598 | 1.418 |
|  | rs2237570 | 1783 | 374 | 28 | 2185 | 0.936 | 0.333 | 0.881 | 0.678 | 1.144 | 1.964 | 0.375 | 0.819 | 0.606 | 1.106 | 1.180 | 0.487 | 2.857 |
| *CDKN1A* | rs1801270 | 1776 | 245 | 22 | 2043 | 0.882 | 0.348 | 0.868 | 0.641 | 1.175 | 0.887 | 0.642 | 0.862 | 0.607 | 1.224 | 0.780 | 0.250 | 2.434 |
|  | rs3176352 | 1080 | 769 | 182 | 2031 | 0.537 | 0.464 | 0.940 | 0.795 | 1.111 | 0.562 | 0.755 | 0.952 | 0.757 | 1.196 | 0.867 | 0.579 | 1.300 |
|  | rs1059234 | 1777 | 243 | 21 | 2041 | 0.719 | 0.397 | 0.879 | 0.649 | 1.192 | 0.738 | 0.692 | 0.869 | 0.612 | 1.233 | 0.828 | 0.265 | 2.582 |
|  | rs6457937 | 1635 | 364 | 30 | 2029 | 0.037 | 0.846 | 0.976 | 0.763 | 1.248 | 0.049 | 0.976 | 0.969 | 0.729 | 1.287 | 0.992 | 0.409 | 2.403 |
|  | rs2395655 | 804 | 1013 | 371 | 2188 | 0.002 | 0.969 | 0.997 | 0.858 | 1.158 | 0.209 | 0.901 | 1.039 | 0.823 | 1.313 | 0.975 | 0.711 | 1.337 |
|  | rs3176331 | 1147 | 856 | 183 | 2186 | 0.762 | 0.383 | 0.928 | 0.785 | 1.098 | 3.749 | 0.153 | 0.810 | 0.645 | 1.018 | 1.042 | 0.716 | 1.515 |
|  | rs3176336 | 1662 | 486 | 37 | 2185 | 0.264 | 0.607 | 1.060 | 0.851 | 1.320 | 0.268 | 0.875 | 1.064 | 0.828 | 1.367 | 1.099 | 0.489 | 2.469 |
|  | rs3176343 | 803 | 996 | 389 | 2188 | 0.365 | 0.545 | 0.955 | 0.823 | 1.108 | 0.662 | 0.718 | 1.004 | 0.796 | 1.266 | 0.891 | 0.651 | 1.220 |
|  | rs3176326 | 1960 | 219 | 10 | 2189 | 3.097 | 0.078 | 0.728 | 0.501 | 1.057 | 3.103 | 0.212 | 0.723 | 0.485 | 1.079 | 0.570 | 0.080 | 4.059 |
| *CDKN1B* | rs34330 | 1137 | 774 | 128 | 2039 | 0.496 | 0.481 | 1.064 | 0.895 | 1.265 | 0.812 | 0.666 | 1.109 | 0.886 | 1.390 | 1.047 | 0.666 | 1.646 |
|  | rs2066827 | 1189 | 732 | 102 | 2023 | 1.603 | 0.205 | 0.887 | 0.735 | 1.070 | 2.598 | 0.273 | 0.826 | 0.654 | 1.044 | 0.943 | 0.575 | 1.546 |
|  | rs7330 | 719 | 952 | 364 | 2035 | 0.139 | 0.709 | 1.029 | 0.885 | 1.197 | 2.170 | 0.338 | 1.178 | 0.925 | 1.499 | 1.005 | 0.727 | 1.389 |
|  | rs3759216 | 665 | 1036 | 446 | 2147 | 0.069 | 0.793 | 0.980 | 0.844 | 1.138 | 0.191 | 0.909 | 0.947 | 0.742 | 1.209 | 0.968 | 0.718 | 1.306 |
|  | rs3759217 | 1696 | 457 | 30 | 2183 | 0.086 | 0.770 | 0.965 | 0.762 | 1.223 | 1.209 | 0.546 | 0.904 | 0.692 | 1.181 | 1.392 | 0.620 | 3.125 |
|  | rs34329 | 1061 | 873 | 242 | 2176 | 0.117 | 0.733 | 1.028 | 0.879 | 1.202 | 0.630 | 0.730 | 1.090 | 0.871 | 1.365 | 0.995 | 0.693 | 1.428 |
|  | rs3093736 | 2043 | 123 | 6 | 2172 | 0.038 | 0.845 | 0.958 | 0.621 | 1.477 | 1.555 | 0.459 | 0.832 | 0.504 | 1.375 | 2.222 | 0.553 | 8.924 |
|  | rs1420023 | 1750 | 388 | 28 | 2166 | 0.007 | 0.932 | 0.989 | 0.775 | 1.263 | 2.420 | 0.298 | 0.886 | 0.667 | 1.179 | 1.685 | 0.796 | 3.568 |

|  | **dbSNP** | **Genotype frequencies** | | | | **Trend test** | | **Risk per allele** | | | **Heterogeneity test** | | **Heterozygote Risk** | | | **Homozygote Risk** | | |
| --- | --- | --- | --- | --- | --- | --- | --- | --- | --- | --- | --- | --- | --- | --- | --- | --- | --- | --- |
| **Gene** | **Reference** | **AA** | **Aa** | **aa** | *Total* | **x^2^** | **P-value** | **HR** | **LCL** | **UCL** | **x^2^** | **P-value** | **HR** | **LCL** | **UCL** | **HR** | **LCL** | **UCL** |
| *CDKN2A* | rs4074785 | 1463 | 489 | 47 | 1999 | 1.226 | 0.268 | 0.879 | 0.697 | 1.109 | 8.422 | 0.015 | 0.704 | 0.526 | 0.942 | 1.520 | 0.851 | 2.714 |
|  | rs3731197 | 1116 | 867 | 194 | 2177 | 0.241 | 0.624 | 0.960 | 0.815 | 1.131 | 0.268 | 0.875 | 0.948 | 0.757 | 1.186 | 0.938 | 0.637 | 1.380 |
|  | rs3731222 | 966 | 916 | 266 | 2148 | 1.378 | 0.241 | 1.097 | 0.941 | 1.279 | 3.311 | 0.191 | 0.969 | 0.769 | 1.222 | 1.301 | 0.951 | 1.780 |
|  | rs3731211 | 1726 | 426 | 38 | 2190 | 0.820 | 0.365 | 0.897 | 0.706 | 1.139 | 1.161 | 0.560 | 0.933 | 0.711 | 1.224 | 0.633 | 0.236 | 1.698 |
|  | rs3218020 | 1760 | 393 | 31 | 2184 | 0.370 | 0.543 | 0.927 | 0.723 | 1.187 | 0.953 | 0.621 | 0.974 | 0.738 | 1.284 | 0.598 | 0.192 | 1.866 |
|  | rs2811712 | 869 | 994 | 298 | 2161 | 2.698 | 0.100 | 1.139 | 0.976 | 1.329 | 4.199 | 0.123 | 1.016 | 0.801 | 1.290 | 1.366 | 1.003 | 1.861 |
|  | rs3218005 | 945 | 941 | 294 | 2180 | 1.702 | 0.192 | 0.901 | 0.770 | 1.055 | 2.151 | 0.341 | 0.852 | 0.679 | 1.070 | 0.855 | 0.608 | 1.200 |
|  | rs3217992 | 1598 | 540 | 52 | 2190 | 0.382 | 0.537 | 1.067 | 0.870 | 1.308 | 0.735 | 0.692 | 1.110 | 0.872 | 1.412 | 0.966 | 0.478 | 1.954 |
|  | rs3731239 | 1833 | 335 | 15 | 2183 | 1.223 | 0.269 | 1.160 | 0.897 | 1.500 | 1.631 | 0.442 | 1.114 | 0.836 | 1.484 | 1.811 | 0.675 | 4.859 |
|  | rs11515 | 2052 | 122 | 2 | 2176 | 0.001 | 0.976 | 1.007 | 0.644 | 1.574 | 1.615 | 0.446 | 0.924 | 0.575 | 1.485 | 4.643 | 0.652 | 33.075 |
|  | rs3088440 | 1208 | 841 | 139 | 2188 | 1.893 | 0.169 | 1.126 | 0.952 | 1.331 | 1.916 | 0.384 | 1.113 | 0.891 | 1.391 | 1.290 | 0.861 | 1.935 |
|  | rs3731249 | 1817 | 357 | 16 | 2190 | 0.641 | 0.423 | 1.111 | 0.861 | 1.434 | 2.121 | 0.346 | 1.028 | 0.772 | 1.369 | 2.079 | 0.859 | 5.034 |
|  | rs3731257 | 816 | 1001 | 343 | 2160 | 0.014 | 0.905 | 0.991 | 0.850 | 1.154 | 2.107 | 0.349 | 0.868 | 0.687 | 1.098 | 1.048 | 0.772 | 1.423 |
| *CDKN2B* | rs1063192 | 700 | 1057 | 424 | 2181 | 1.003 | 0.317 | 0.926 | 0.796 | 1.077 | 3.998 | 0.135 | 0.786 | 0.620 | 0.997 | 0.908 | 0.676 | 1.220 |
|  | rs3218009 | 1660 | 484 | 32 | 2176 | 0.190 | 0.663 | 0.950 | 0.754 | 1.198 | 1.125 | 0.570 | 0.897 | 0.691 | 1.164 | 1.300 | 0.579 | 2.920 |
|  | rs3218012 | 710 | 1039 | 427 | 2176 | 3.248 | 0.072 | 1.147 | 0.988 | 1.331 | 7.075 | 0.029 | 0.938 | 0.732 | 1.201 | 1.345 | 1.013 | 1.787 |
| *CDKN2C* | rs12855 | 1798 | 350 | 21 | 2169 | 2.179 | 0.140 | 1.208 | 0.946 | 1.543 | 2.179 | 0.336 | 1.210 | 0.920 | 1.592 | 1.443 | 0.538 | 3.873 |
|  | rs3176459 | 946 | 966 | 255 | 2167 | 0.151 | 0.697 | 1.032 | 0.881 | 1.208 | 0.817 | 0.665 | 0.964 | 0.768 | 1.210 | 1.128 | 0.806 | 1.579 |
| *CDKN2D* | rs1465701 | 1958 | 206 | 7 | 2171 | 0.257 | 0.612 | 0.913 | 0.638 | 1.306 | 0.280 | 0.869 | 0.904 | 0.618 | 1.323 | 0.964 | 0.135 | 6.868 |
|  | rs3218222 | 1254 | 803 | 125 | 2182 | 0.019 | 0.892 | 0.988 | 0.828 | 1.179 | 4.069 | 0.131 | 0.849 | 0.674 | 1.070 | 1.283 | 0.853 | 1.929 |

**Supplementary table 2. All-cause mortality hazard ratios associated with SNPs tagged by multimarker tagSNPs haplotypes**

| **Gene** | **Multimarker**  **TagSNPs** | **SNPs tagged** | **Haplotype** | **Frequency** | **OR (95% CI)** | **P** |
| --- | --- | --- | --- | --- | --- | --- |
| *CDK6* | rs4729049, rs992519 | rs2374594  rs6975474  rs2374589  rs10246604 | 11 | 0.06 | 1.25 (0.92-1.68) | 0.15 |
| *CCND2* | rs3217820, rs3217862 | rs3217827  rs3217881 | 00 | 0.46 | 1.01 (0.87-1.18) | 0.87 |
|  | rs3217869, rs3217852 | rs3217830  rs3217896 | 00 | 0.38 | 1.04 (0.89-1.22) | 0.62 |
|  | rs3217926, rs3217936 | rs4625554 | 00 | 0.28 | 0.93 (0.78-1.10) | 0.38 |
|  | rs3217926, rs3217925,  rs3217916 | rs3217907 | 000 | 0.33 | 0.98 (0.84-1.16) | 0.85 |
