# Supplementary material for: Effects of common germline genetic variation in cell cycle control genes on breast cancer survival: results from a population-based cohort
Source: Breast Cancer Res. 2008 May 28;10(3):R47. doi: 10.1186/bcr2100 (PMC2481496; doi:10.1186/bcr2100)
Supplement: Additional File 2 — This file contains Supplementary tables 3 and 4, which show the results of the univariate breast cancer specific mortality Cox regression analyses for single marker tagSNPs and multimarker tagSNPs. [file bcr2100-S2.doc]

Supplementary table 3. Breast cancer specific mortality hazard ratios associated with common tagSNPs in cell cycle genes after a diagnosis of breast cancer

|  | **dbSNP** | **Genotype frequencies** | | | | **Trend test** | | **Risk per allele** | | | **Heterogeneity test** | | **Heterozygote Risk** | | | **Homozygote Risk** | | |
| --- | --- | --- | --- | --- | --- | --- | --- | --- | --- | --- | --- | --- | --- | --- | --- | --- | --- | --- |
| **Gene** | **Reference** | **AA** | **Aa** | **aa** | *Total* | **x^2^** | **P-value** | **HR** | **LCL** | **UCL** | **x^2^** | **P-value** | **HR** | **LCL** | **UCL** | **HR** | **LCL** | **UCL** |
| *CCND1* | rs7178 | 1744 | 282 | 12 | 2038 | 0.314 | 0.575 | 1.090 | 0.809 | 1.468 | 1.093 | 0.579 | 1.149 | 0.835 | 1.580 | 0.580 | 0.081 | 4.134 |
|  | rs3862792 | 1917 | 122 | 1 | 2040 | 0.948 | 0.330 | 1.253 | 0.807 | 1.946 | 2.594 | 0.273 | 1.154 | 0.724 | 1.839 | 7.361 | 1.032 | 52.518 |
|  | rs603965 | 627 | 982 | 428 | 2037 | 0.015 | 0.902 | 0.990 | 0.840 | 1.166 | 0.018 | 0.991 | 0.995 | 0.761 | 1.302 | 0.978 | 0.703 | 1.362 |
|  | rs678653 | 811 | 946 | 263 | 2020 | 0.041 | 0.840 | 1.018 | 0.855 | 1.212 | 1.774 | 0.412 | 0.898 | 0.696 | 1.159 | 1.132 | 0.792 | 1.616 |
|  | rs602652 | 612 | 1057 | 502 | 2171 | 0.085 | 0.770 | 0.976 | 0.830 | 1.148 | 0.806 | 0.668 | 1.073 | 0.816 | 1.410 | 0.941 | 0.674 | 1.314 |
|  | rs3212879 | 614 | 1076 | 483 | 2173 | 0.034 | 0.853 | 0.985 | 0.836 | 1.159 | 0.075 | 0.963 | 0.963 | 0.734 | 1.262 | 0.973 | 0.702 | 1.348 |
|  | rs3212891 | 692 | 1067 | 431 | 2190 | 0.077 | 0.781 | 0.977 | 0.829 | 1.151 | 0.213 | 0.899 | 0.940 | 0.724 | 1.221 | 0.965 | 0.694 | 1.341 |
| *CCND2* | rs3217795 | 1756 | 409 | 21 | 2186 | 0.274 | 0.601 | 0.930 | 0.706 | 1.225 | 2.961 | 0.227 | 0.828 | 0.606 | 1.132 | 1.776 | 0.733 | 4.308 |
|  | rs3217805 | 812 | 1021 | 330 | 2163 | 0.238 | 0.626 | 1.042 | 0.883 | 1.229 | 2.483 | 0.289 | 1.205 | 0.935 | 1.554 | 1.004 | 0.697 | 1.444 |
|  | rs3217820 | 842 | 1056 | 283 | 2181 | 0.266 | 0.606 | 1.046 | 0.882 | 1.240 | 6.208 | 0.045 | 0.827 | 0.642 | 1.064 | 1.248 | 0.897 | 1.738 |
|  | rs3217869 | 808 | 1050 | 325 | 2183 | 0.047 | 0.829 | 0.982 | 0.829 | 1.162 | 7.934 | 0.019 | 0.745 | 0.578 | 0.961 | 1.111 | 0.805 | 1.535 |
|  | rs3217926 | 794 | 1061 | 330 | 2185 | 0.518 | 0.472 | 1.064 | 0.900 | 1.258 | 3.577 | 0.167 | 0.892 | 0.691 | 1.153 | 1.218 | 0.878 | 1.689 |
|  | rs3217936 | 1004 | 936 | 247 | 2187 | 1.034 | 0.309 | 1.092 | 0.922 | 1.293 | 3.622 | 0.163 | 0.938 | 0.730 | 1.205 | 1.329 | 0.941 | 1.877 |
|  | rs3217852 | 1283 | 778 | 128 | 2189 | 0.395 | 0.530 | 0.940 | 0.776 | 1.140 | 0.794 | 0.672 | 0.987 | 0.773 | 1.260 | 0.788 | 0.457 | 1.358 |
|  | rs3217862 | 1511 | 605 | 71 | 2187 | 0.335 | 0.563 | 0.938 | 0.753 | 1.168 | 13.052 | 0.001 | 0.692 | 0.521 | 0.920 | 1.806 | 1.102 | 2.959 |
|  | rs3217863 | 1874 | 310 | 9 | 2193 | 0.570 | 0.450 | 0.882 | 0.633 | 1.229 | 4.126 | 0.127 | 0.773 | 0.535 | 1.116 | 2.576 | 0.825 | 8.042 |
|  | rs3217906 | 1214 | 814 | 157 | 2185 | 0.001 | 0.979 | 1.002 | 0.833 | 1.206 | 10.807 | 0.005 | 0.748 | 0.578 | 0.968 | 1.462 | 0.997 | 2.143 |
|  | rs3217916 | 1144 | 879 | 165 | 2188 | 0.495 | 0.482 | 1.067 | 0.891 | 1.278 | 4.174 | 0.124 | 0.904 | 0.706 | 1.158 | 1.394 | 0.947 | 2.052 |
|  | rs3217925 | 1213 | 825 | 133 | 2171 | 0.051 | 0.821 | 0.978 | 0.809 | 1.183 | 1.403 | 0.496 | 0.891 | 0.696 | 1.141 | 1.137 | 0.721 | 1.791 |
|  | rs3217933 | 1226 | 826 | 136 | 2188 | 1.794 | 0.180 | 0.877 | 0.722 | 1.065 | 1.985 | 0.371 | 0.848 | 0.663 | 1.085 | 0.831 | 0.498 | 1.386 |
|  | rs3217901 | 745 | 1074 | 371 | 2190 | 0.129 | 0.720 | 1.031 | 0.874 | 1.215 | 0.180 | 0.914 | 1.054 | 0.815 | 1.364 | 1.053 | 0.748 | 1.481 |
| *CCND3* | rs1410492 | 1191 | 847 | 146 | 2184 | 2.477 | 0.116 | 0.858 | 0.707 | 1.041 | 2.667 | 0.264 | 0.886 | 0.696 | 1.129 | 0.678 | 0.393 | 1.170 |
|  | rs2479717 | 2374 | 1674 | 313 | 4361 | 11.953 | **0.001** | 1.263 | 1.109 | 1.438 | 12.017 | 0.002 | 1.247 | 1.040 | 1.494 | 1.462 | 1.075 | 1.988 |
|  | rs1051130 | 715 | 1015 | 458 | 2188 | 1.362 | 0.243 | 1.098 | 0.938 | 1.285 | 1.993 | 0.369 | 1.199 | 0.916 | 1.569 | 1.192 | 0.863 | 1.647 |
|  | rs3218092 | 1498 | 636 | 57 | 2191 | 2.258 | 0.133 | 0.842 | 0.669 | 1.059 | 2.260 | 0.323 | 0.844 | 0.649 | 1.099 | 0.698 | 0.310 | 1.571 |
|  | rs9529 | 1173 | 849 | 161 | 2183 | 5.303 | **0.021** | 1.231 | 1.034 | 1.466 | 5.937 | 0.051 | 1.317 | 1.035 | 1.677 | 1.393 | 0.916 | 2.119 |
|  | rs3218110 | 1216 | 825 | 142 | 2183 | 0.342 | 0.559 | 1.056 | 0.880 | 1.269 | 6.802 | 0.033 | 1.281 | 1.011 | 1.623 | 0.722 | 0.410 | 1.272 |
|  | rs3218114 | 1490 | 641 | 53 | 2184 | 1.892 | 0.169 | 0.854 | 0.678 | 1.074 | 2.028 | 0.363 | 0.874 | 0.673 | 1.133 | 0.635 | 0.262 | 1.542 |
| *CCNE1* | rs997669 | 761 | 1061 | 361 | 2183 | 1.601 | 0.206 | 1.113 | 0.943 | 1.313 | 1.798 | 0.407 | 1.063 | 0.819 | 1.379 | 1.257 | 0.902 | 1.752 |
|  | rs3218038 | 2027 | 157 | 1 | 2185 | . | . | . | . | . | . | . | . | . | . | . | . | . |
|  | rs3218076 | 1170 | 880 | 137 | 2187 | 0.115 | 0.734 | 0.968 | 0.801 | 1.169 | 0.154 | 0.926 | 0.982 | 0.773 | 1.249 | 0.907 | 0.550 | 1.496 |
|  | rs3218036 | 987 | 956 | 243 | 2186 | 1.544 | 0.214 | 1.115 | 0.940 | 1.321 | 2.519 | 0.284 | 1.017 | 0.794 | 1.304 | 1.332 | 0.934 | 1.902 |

|  | **dbSNP** | **Genotype frequencies** | | | | **Trend test** | | **Risk per allele** | | | **Heterogeneity test** | | **Heterozygote Risk** | | | **Homozygote Risk** | | |
| --- | --- | --- | --- | --- | --- | --- | --- | --- | --- | --- | --- | --- | --- | --- | --- | --- | --- | --- |
| **Gene** | **Reference** | **AA** | **Aa** | **aa** | *Total* | **x^2^** | **P-value** | **HR** | **LCL** | **UCL** | **x^2^** | **P-value** | **HR** | **LCL** | **UCL** | **HR** | **LCL** | **UCL** |
| *CDK2* | rs2069408 | 971 | 964 | 239 | 2174 | 0.014 | 0.907 | 0.990 | 0.833 | 1.176 | 0.158 | 0.924 | 1.023 | 0.802 | 1.306 | 0.947 | 0.640 | 1.401 |
|  | rs1045435 | 1838 | 326 | 20 | 2184 | 1.438 | 0.230 | 1.186 | 0.904 | 1.556 | 1.505 | 0.471 | 1.208 | 0.890 | 1.638 | 1.238 | 0.396 | 3.867 |
| *CDK4* | rs2270777 | 630 | 1028 | 383 | 2041 | 0.316 | 0.574 | 1.050 | 0.887 | 1.242 | 0.361 | 0.835 | 1.026 | 0.783 | 1.344 | 1.107 | 0.790 | 1.553 |
| *CDK6* | rs3731343 | 619 | 1083 | 466 | 2168 | 2.063 | 0.151 | 0.887 | 0.752 | 1.045 | 3.289 | 0.193 | 0.995 | 0.764 | 1.296 | 0.760 | 0.537 | 1.074 |
|  | rs3757823 | 1751 | 410 | 18 | 2179 | 0.302 | 0.583 | 1.078 | 0.827 | 1.405 | 2.278 | 0.320 | 0.982 | 0.728 | 1.323 | 2.135 | 0.880 | 5.179 |
|  | rs2079147 | 1227 | 2126 | 996 | 4349 | 1.95† | 0.052 | 1.147 | 0.999 | 1.317 | 5.235 | 0.073 | 1.024 | 0.829 | 1.266 | 1.230 | 0.968 | 1.564 |
|  | rs4729049 | 1803 | 364 | 14 | 2181 | 0.390 | 0.532 | 1.094 | 0.828 | 1.445 | 0.407 | 0.816 | 1.102 | 0.816 | 1.488 | 1.104 | 0.274 | 4.440 |
|  | rs8 | 1349 | 700 | 88 | 2137 | 0.261 | 0.610 | 0.947 | 0.768 | 1.168 | 3.496 | 0.174 | 0.824 | 0.635 | 1.069 | 1.293 | 0.764 | 2.188 |
|  | rs445 | 1735 | 412 | 24 | 2171 | 0.496 | 0.481 | 1.099 | 0.848 | 1.423 | 1.421 | 0.491 | 1.029 | 0.768 | 1.379 | 1.797 | 0.741 | 4.361 |
|  | rs992519 | 1614 | 512 | 47 | 2173 | 0.361 | 0.548 | 1.073 | 0.854 | 1.349 | 0.482 | 0.786 | 1.100 | 0.843 | 1.435 | 1.027 | 0.456 | 2.312 |
|  | rs42046 | 1141 | 874 | 152 | 2167 | 2.732 | 0.098 | 1.165 | 0.974 | 1.394 | 3.127 | 0.209 | 1.227 | 0.964 | 1.562 | 1.258 | 0.809 | 1.957 |
|  | rs8179 | 1325 | 724 | 96 | 2145 | 0.982 | 0.322 | 1.105 | 0.909 | 1.344 | 3.136 | 0.208 | 1.232 | 0.966 | 1.572 | 0.902 | 0.490 | 1.662 |
|  | rs2282991 | 1833 | 332 | 13 | 2178 | 0.353 | 0.552 | 1.094 | 0.817 | 1.464 | 0.477 | 0.788 | 1.072 | 0.784 | 1.465 | 1.520 | 0.378 | 6.113 |
|  | rs3731348 | 1914 | 252 | 3 | 2169 | . | . | . | . | . | . | . | . | . | . | . | . | . |
|  | rs2285332 | 1245 | 783 | 151 | 2179 | 0.063 | 0.802 | 1.024 | 0.853 | 1.228 | 0.095 | 0.954 | 1.009 | 0.789 | 1.289 | 1.073 | 0.688 | 1.674 |
|  | rs2237570 | 1783 | 374 | 28 | 2185 | 0.508 | 0.476 | 0.904 | 0.682 | 1.198 | 2.170 | 0.338 | 0.815 | 0.587 | 1.130 | 1.398 | 0.577 | 3.389 |
| *CDKN1A* | rs1801270 | 1776 | 245 | 22 | 2043 | 1.808 | 0.179 | 0.797 | 0.566 | 1.124 | 1.815 | 0.403 | 0.804 | 0.544 | 1.187 | 0.604 | 0.150 | 2.429 |
|  | rs3176352 | 1080 | 769 | 182 | 2031 | 0.726 | 0.394 | 0.925 | 0.771 | 1.109 | 1.511 | 0.470 | 0.996 | 0.779 | 1.273 | 0.759 | 0.476 | 1.210 |
|  | rs1059234 | 1777 | 243 | 21 | 2041 | 1.597 | 0.206 | 0.807 | 0.572 | 1.139 | 1.597 | 0.450 | 0.810 | 0.548 | 1.196 | 0.640 | 0.159 | 2.575 |
|  | rs6457937 | 1635 | 364 | 30 | 2029 | 0.016 | 0.900 | 0.983 | 0.754 | 1.283 | 0.245 | 0.885 | 0.946 | 0.693 | 1.291 | 1.162 | 0.479 | 2.819 |
|  | rs2395655 | 804 | 1013 | 371 | 2188 | 0.253 | 0.615 | 0.959 | 0.814 | 1.130 | 0.332 | 0.847 | 0.933 | 0.724 | 1.201 | 0.932 | 0.664 | 1.308 |
|  | rs3176331 | 1147 | 856 | 183 | 2186 | 1.925 | 0.165 | 0.879 | 0.731 | 1.057 | 5.089 | 0.078 | 0.755 | 0.588 | 0.970 | 0.965 | 0.639 | 1.459 |
|  | rs3176336 | 1662 | 486 | 37 | 2185 | 0.129 | 0.719 | 1.045 | 0.822 | 1.329 | 0.131 | 0.937 | 1.048 | 0.797 | 1.377 | 1.079 | 0.444 | 2.617 |
|  | rs3176343 | 803 | 996 | 389 | 2188 | 1.726 | 0.189 | 0.897 | 0.762 | 1.056 | 1.730 | 0.421 | 0.892 | 0.694 | 1.146 | 0.807 | 0.573 | 1.135 |
|  | rs3176326 | 1960 | 219 | 10 | 2189 | 1.31† | 0.191 | 0.734 | 0.461 | 1.168 | 4.879 | 0.087 | 0.634 | 0.398 | 1.009 | 0.705 | 0.099 | 5.029 |
| *CDKN1B* | rs34330 | 1137 | 774 | 128 | 2039 | 1.230 | 0.267 | 1.112 | 0.923 | 1.338 | 2.599 | 0.273 | 1.219 | 0.957 | 1.553 | 1.035 | 0.626 | 1.710 |
|  | rs2066827 | 1189 | 732 | 102 | 2023 | 1.828 | 0.176 | 0.870 | 0.708 | 1.068 | 2.633 | 0.268 | 0.812 | 0.629 | 1.047 | 0.906 | 0.526 | 1.562 |
|  | rs7330 | 719 | 952 | 364 | 2035 | 0.854 | 0.355 | 1.080 | 0.918 | 1.271 | 3.121 | 0.210 | 1.264 | 0.970 | 1.646 | 1.108 | 0.782 | 1.570 |
|  | rs3759216 | 665 | 1036 | 446 | 2147 | 0.981 | 0.322 | 0.921 | 0.782 | 1.084 | 0.998 | 0.607 | 0.934 | 0.718 | 1.214 | 0.845 | 0.605 | 1.180 |
|  | rs3759217 | 1696 | 457 | 30 | 2183 | 0.073 | 0.788 | 1.035 | 0.806 | 1.330 | 1.477 | 0.478 | 0.954 | 0.716 | 1.271 | 1.672 | 0.743 | 3.762 |
|  | rs34329 | 1061 | 873 | 242 | 2176 | 0.510 | 0.475 | 1.064 | 0.898 | 1.260 | 0.655 | 0.721 | 1.101 | 0.861 | 1.408 | 1.096 | 0.749 | 1.604 |
|  | rs3093736 | 2043 | 123 | 6 | 2172 | 0.009 | 0.923 | 1.023 | 0.648 | 1.615 | 1.699 | 0.428 | 0.868 | 0.507 | 1.485 | 2.650 | 0.659 | 10.652 |
|  | rs1420023 | 1750 | 388 | 28 | 2166 | 0.049 | 0.826 | 0.970 | 0.742 | 1.268 | 4.352 | 0.113 | 0.820 | 0.596 | 1.128 | 1.971 | 0.929 | 4.180 |

|  | **dbSNP** | **Genotype frequencies** | | | | **Trend test** | | **Risk per allele** | | | **Heterogeneity test** | | **Heterozygote Risk** | | | **Homozygote Risk** | | |
| --- | --- | --- | --- | --- | --- | --- | --- | --- | --- | --- | --- | --- | --- | --- | --- | --- | --- | --- |
| **Gene** | **Reference** | **AA** | **Aa** | **aa** | *Total* | **x^2^** | **P-value** | **HR** | **LCL** | **UCL** | **x^2^** | **P-value** | **HR** | **LCL** | **UCL** | **HR** | **LCL** | **UCL** |
| *CDKN2A* | rs4074785 | 1463 | 489 | 47 | 1999 | 1.618 | 0.203 | 0.850 | 0.659 | 1.098 | 8.138 | 0.017 | 0.675 | 0.491 | 0.930 | 1.474 | 0.781 | 2.781 |
|  | rs3731197 | 1116 | 867 | 194 | 2177 | 0.136 | 0.712 | 0.967 | 0.809 | 1.156 | 0.203 | 0.903 | 0.946 | 0.741 | 1.208 | 0.963 | 0.636 | 1.460 |
|  | rs3731222 | 966 | 916 | 266 | 2148 | 2.518 | 0.113 | 1.145 | 0.970 | 1.352 | 4.825 | 0.090 | 0.986 | 0.765 | 1.271 | 1.426 | 1.021 | 1.990 |
|  | rs3731211 | 1726 | 426 | 38 | 2190 | 0.331 | 0.565 | 0.928 | 0.719 | 1.199 | 1.150 | 0.563 | 0.990 | 0.741 | 1.323 | 0.568 | 0.182 | 1.773 |
|  | rs3218020 | 1760 | 393 | 31 | 2184 | 0.135 | 0.713 | 0.952 | 0.729 | 1.242 | 1.458 | 0.482 | 1.028 | 0.765 | 1.382 | 0.478 | 0.119 | 1.921 |
|  | rs2811712 | 869 | 994 | 298 | 2161 | 1.76† | 0.078 | 1.186 | 0.981 | 1.435 | 6.651 | 0.036 | 0.988 | 0.729 | 1.338 | 1.493 | 1.032 | 2.161 |
|  | rs3218005 | 945 | 941 | 294 | 2180 | 0.670 | 0.413 | 0.932 | 0.786 | 1.104 | 0.792 | 0.673 | 0.902 | 0.705 | 1.156 | 0.893 | 0.617 | 1.290 |
|  | rs3217992 | 1598 | 540 | 52 | 2190 | 0.737 | 0.391 | 1.102 | 0.885 | 1.372 | 1.134 | 0.567 | 1.153 | 0.889 | 1.495 | 1.010 | 0.475 | 2.145 |
|  | rs3731239 | 1833 | 335 | 15 | 2183 | 0.705 | 0.401 | 1.131 | 0.853 | 1.500 | 0.870 | 0.647 | 1.101 | 0.805 | 1.505 | 1.588 | 0.509 | 4.959 |
|  | rs11515 | 2052 | 122 | 2 | 2176 | 0.043 | 0.835 | 0.949 | 0.576 | 1.562 | 2.158 | 0.340 | 0.845 | 0.494 | 1.446 | 5.421 | 0.761 | 38.641 |
|  | rs3088440 | 1208 | 841 | 139 | 2188 | 1.400 | 0.237 | 1.117 | 0.931 | 1.340 | 1.675 | 0.433 | 1.070 | 0.839 | 1.364 | 1.333 | 0.867 | 2.051 |
|  | rs3731249 | 1817 | 357 | 16 | 2190 | 0.038 | 0.844 | 1.029 | 0.773 | 1.370 | 1.569 | 0.456 | 0.943 | 0.684 | 1.300 | 1.936 | 0.721 | 5.200 |
|  | rs3731257 | 816 | 1001 | 343 | 2160 | 0.114 | 0.735 | 0.972 | 0.822 | 1.148 | 3.786 | 0.151 | 0.803 | 0.622 | 1.037 | 1.039 | 0.748 | 1.443 |
| *CDKN2B* | rs1063192 | 700 | 1057 | 424 | 2181 | 1.544 | 0.214 | 0.901 | 0.764 | 1.063 | 4.138 | 0.126 | 0.765 | 0.592 | 0.989 | 0.865 | 0.626 | 1.194 |
|  | rs3218009 | 1660 | 484 | 32 | 2176 | 0.154 | 0.695 | 0.951 | 0.739 | 1.223 | 0.889 | 0.641 | 0.900 | 0.678 | 1.194 | 1.289 | 0.531 | 3.127 |
|  | rs3218012 | 710 | 1039 | 427 | 2176 | 3.236 | 0.072 | 1.160 | 0.987 | 1.364 | 6.869 | 0.032 | 0.936 | 0.714 | 1.227 | 1.376 | 1.011 | 1.871 |
| *CDKN2C* | rs12855 | 1798 | 350 | 21 | 2169 | 0.844 | 0.358 | 1.139 | 0.867 | 1.496 | 0.847 | 0.655 | 1.143 | 0.844 | 1.548 | 1.262 | 0.404 | 3.944 |
|  | rs3176459 | 946 | 966 | 255 | 2167 | 0.032 | 0.859 | 1.016 | 0.855 | 1.206 | 0.403 | 0.817 | 0.962 | 0.752 | 1.230 | 1.083 | 0.748 | 1.568 |
| *CDKN2D* | rs1465701 | 1958 | 206 | 7 | 2171 | . | . | . | . | . | . | . | . | . | . | . | . | . |
|  | rs3218222 | 1254 | 803 | 125 | 2182 | 0.236 | 0.627 | 0.953 | 0.785 | 1.157 | 4.857 | 0.088 | 0.799 | 0.620 | 1.030 | 1.255 | 0.806 | 1.955 |

**Supplementary table 4. Breast cancer specific mortality hazard ratios associated with SNPs tagged by multimarker tagSNPs haplotypes**

| **Gene** | **Multimarker**  **TagSNPs** | **SNPs tagged** | **Haplotype** | **Frequency** | **OR (95% CI)** | **P** |
| --- | --- | --- | --- | --- | --- | --- |
| *CDK6* | rs4729049, rs992519 | rs2374594  rs6975474  rs2374589  rs10246604 | 11 | 0.06 | 1.21 (0.87-1.68) | 0.26 |
| *CCND2* | rs3217820, rs3217862 | rs3217827  rs3217881 | 00 | 0.46 | 0.99 (0.84-1.17) | 0.90 |
|  | rs3217869, rs3217852 | rs3217830  rs3217896 | 00 | 0.38 | 1.06 (0.90-1.26) | 0.47 |
|  | rs3217926, rs3217936 | rs4625554 | 00 | 0.28 | 0.84 (0.69-1.01) | 0.07 |
|  | rs3217926, rs3217925,  rs3217916 | rs3217907 | 000 | 0.33 | 0.90 (0.75-1.08) | 0.25 |
