# Supplementary material for: Effects of common germline genetic variation in cell cycle control genes on breast cancer survival: results from a population-based cohort
Source: Breast Cancer Res. 2008 May 28;10(3):R47. doi: 10.1186/bcr2100 (PMC2481496; doi:10.1186/bcr2100)
Supplement: Additional File 3 — This file contains Supplementary tables 5 and 6, which show additional information regarding the microarray datasets used in the somatic expression analyses. Supplementary table 5 shows additional microarray study and patient characteristics. Supplementary table 6 shows hazard ratios associated with microarray expression of genes in linkage disequilibrium with CCND3 rs2479717. [file bcr2100-S3.doc]

**Supplementary table 5. Microarray study patient and tumor** characteristics

|  |  | **Grade** | | | | **ER Status** | | |
| --- | --- | --- | --- | --- | --- | --- | --- | --- |
| **Study (ref)** | **Median**  **Diagnosis Age** | **1** | **2** | **3** | **Missing** | **-** | **+** | **Missing** |
| Blenkiron, et al. (22) | 59 | 33 | 52 | 43 | 0 | 40 | 88 | 0 |
| Chin, et al. (23) | 55 | 14 | 46 | 64 | 5 | 46 | 83 | 0 |
| Miller, et al. (24) | 65 | 62 | 121 | 51 | 0 | 31 | 201 | 2 |
| Sorlie, et al. (25) | 57 | 9 | 33 | 33 | 1 | 18 | 56 | 2 |
| Sotitiou, et al. (26) | 60 | 24 | 20 | 32 | 18 | 24 | 65 | 5 |
| van deVijver, et al. (27) | <53* | 75 | 101 | 119 | 0 | 69 | 226 | 0 |
| Wang, et al. (28) | 54*† | N\A | N\A | N\A | N\A | 77 | 208 | 0 |
| ALL |  | 217 | 373 | 342 | 24 | 305 | 927 | 9 |

* Age cited from publication, not calculated.

† Mean age

**Supplementary table 6. Hazard ratios associated with microarray expression of genes in linkage disequilibrium with *CCND3* rs2479717**

|  |  | **Fixed Effects Analysis*** | | **Random Effects Analysis†** | |
| --- | --- | --- | --- | --- | --- |
| **Gene** | **N** | **HR (95% CI)** | **P** | **HR (95% CI)** | **P** |
| *PGC* | 1165 | 1.04 (0.85-1.28) | 0.71 | 1.04 (0.85-1.28) | 0.71 |
| *FRS3* | 1165 | 0.92 (0.61-1.36) | 0.66 | 0.99 (0.57-1.70) | 0.96 |
| *C6orf49* | 1071 | **1.60 (1.18-2.16)** | **0.002** | **1.84 (1.11-3.05)** | **0.02** |
| *BYSL* | 1241 | 1.17 (0.99-1.38) | 0.08 | **1.84 (1.10-3.08)** | **0.02** |
| *CCND3* | 1220 | 0.82 (0.63-1.07) | 0.15 | 0.82 (0.63-1.07) | 0.15 |
| *USP49* | 499 | 1.10 (0.63-1.92) | 0.74 | 1.10 (0.63-1.92) | 0.74 |
| *TRFP* | 636 | 0.84 (0.69-1.02) | 0.08 | 0.84 (0.69-1.02) | 0.08 |

* Fixed effects meta-analysis

† Random effects meta-analysis
